# Domestic

**Published:** 1841-04-17

**Authors:** 


### DOMESTIC.

### HEALTH OF THE CITY.

INTERMENTS *in the City and Liberties of Philadelphia, from the 3d to the 10th of April,*

| Diseases. | Adults. | Children. |
|---|---|---|
| Asthma, | 1 | 0 |
| Amennorrhœa, | 1 | 0 |
| Casualty, | 1 | 0 |
| Congestion of the lungs, | 0 | 1 |
| —— brain, | 0 | 1 |
| Consumption of the lungs, | 22 | 5 |
| Convulsions, | 0 | 5 |
| Constipation, | 1 | 0 |
| Dropsy, | 1 | 0 |
| —— abdominal, | 1 | 0 |
| —— head, | 0 | 3 |
| —— breast, | 1 | 0 |
| Disease of brain, | 1 | 0 |
| —— heart, | 2 | 0 |
| —— lungs, | 0 | 1 |
| —— hip-joint, | 0 | 1 |
| Dysentery, | 0 | 1 |
| Debility, | 0 | 8 |
| Erysipelas, | 2 | 0 |
| Fever, | 0 | 1 |
| —— scarlet, | 0 | 1 |
| —— typhus, | 2 | 0 |
| Hooping cough, | 0 | 2 |
| Intestinal irritation, | 0 | 1 |
| Inflammation of the brain, | 0 | 2 |
| —— bronchi, | 1 | 3 |
| —— lungs, | 3 | 5 |
| —— bowels, | 1 | 1 |
| —— heart, | 1 | 0 |
| —— uterus, | 0 | 1 |
| *Carried forward,* | 42 | 43 |
| *Brought forward,* | 42 | 43 |
| Intemperance and exposure, | 3 | 0 |
| Marasmus, | 0 | 1 |
| Measles, | 0 | 5 |
| Mania a Potu, | 1 | 0 |
| Old age, | 4 | 0 |
| Obstruction of bowels, | 0 | 1 |
| Pleurisy, | 1 | 0 |
| Scirrhus of liver, | 1 | 0 |
| Small pox, | 1 | 3 |
| Still-born, | 0 | 8 |
| Ulceration of throat, | 1 | 0 |
| Unknown, | 3 | 0 |
| Varioloid, | 0 | 1 |
| Total, 119— | 57 | 62 |

Of the above, there

| | |
|---|---|
| were under 1 year | 28 |
| From 1 to 2 | 9 |
| 2 to 5 | 13 |
| 5 to 10 | 7 |
| 10 to 15 | 2 |
| 15 to 20 | 3 |
| 20 to 30 | 10 |
| 30 to 40 | 17 |
| 40 to 50 | 7 |
| 50 to 60 | 7 |
| 60 to 70 | 7 |
| 70 to 80 | 6 |
| 80 to 90 | 3 |
| Total, | 119 |

In the above are included 17 people of colour, 6 interments from the alms-house, and 1 from the country.

*Cases of Thymic Asthma.*—S. P. W. aged sixteen months. Soon after birth, before the child was dressed, he had a cough, which was repeated occasionally, during the rest of his life. It was noticed less, perhaps, during the last few months. In April last, when five and a half months old, he appeared rather delicate and feeble. He appeared to increase in length, but remained stationary in flesh.

When eight months old he was attacked with spasms at midnight. His mother was awoke with a noise resembling a hiccup.—When taken up, he appeared like a child partly insensible. There was laboured respiration, and stiffness of the extremities, with lividness of the lips and nose, and the eyes were fixed. The paroxysm passed off in about two minutes, and left him with a twitching of the extremities, which lasted about five minutes. These paroxysms returned twice with the interval of a week. The last one was the least severe.

When nine months old, while travelling, he appeared to have taken cold; and at Pittsfield, Mass., they supposed his symptoms to be those of pneumonia. His attending physician, Dr. Root, administered powders composed of calomel and ipecac, and applied a blister plaster to the chest. This treatment had a favourable effect, and he was soon relieved.

From that time, unil he was fourteen and a half months old, he appeared well and grew rapidly, notwithstanding he never had teeth. He was not able to walk without assistance, which was attributed to a weakness of the limbs.

When fourteen and a half months old, he was attacked with convulsions attended with spasms of the muscles of the face and eyes, frothing of the mouth, and sickness of the stomach. The next day there were symptoms of bronchitis, and a slight catching of the breath, of an asthmatic character, which had troubled him also from the sixth to the tenth month of his age. This catching of the breath appeared to be relieved by the treatment at Pittsfield.—The two following days he had a return of convulsions which were not so severe as the first.

These convulsions were followed by cough and frequent respiration, which were relieved by calomel and ipecac, an enema, and a blister plaster to the chest.

From that time until the week before his death, he appeared perfectly well, except that a few days before his death the catching of the breath returned.

Five days previous, he appeared to have lost his breath entirely, and became very dark coloured in the face. When the breathing returned he evinced distress by crying.

Four days previous, he had a convulsion, preceded by the catching of the breath. At the time of his death this "catching of the breath" appeared, while amusing himself with his playthings, and he expired in three minutes.

*Post-mortem examination* was conducted by Dr. A. C. Post, about seven hours after death. Body well formed and developed. On raising the sternum, the thymus gland was brought into view, appearing at first, rather under the natural size at birth, being about one and a half inches in length. But on more careful examination, this was found to be but a kind of appendix to, or prolongation of, the true body of the gland, which was situated higher up upon the anterior portion of the trachea: it was two and a half inches long and one and a half broad, and about half an inch at its thickest part.—The right cornu was natural, but the left extended upwards by a worm-like process, quite to the angle of the jaw, and terminated in a bulb as large as the end of the little finger. It lay directly upon the track of the great vessels passing down the neck. The vena innominata also lay enclosed between the body of the gland and the lower prolongation, or lobe;—but the par vagum of the phrenic nerves were not implicated by the tumour. The whole length of the gland when removed from its attachments was six inches. There was no enlargement of the lymphatic glands of the neck.—The heart was generally enlarged. Both sides; especially the auricles, as also the great vessels, were considerably distended by very dark and fluid blood. The mucous glands about the pharynx and root of the tongue were enlarged.

The larynx, trachea, bronchi and lungs were perfectly healthy; no hypostatic congestion.

*A case of enlargement of the thymus gland, occurring in a child nineteen months old, and terminating fatally in nine days.*—Isaiah Harris, aged 19 months, a light mulatto, was attacked on Thursday, the 18th Feb., with fever. When I saw him a day or two afterwards, he had a hot skin, a loaded tongue, a frequent pulse, quick breathing, and green stools; he was rather hoarse, and had a short, stifled, harsh cough. From his excessive peevishness it was impossible to auscultate the chest. On the cheeks were clusters of reddish papulæ, and all over the body a fine granular eruption without redness, and resembling the whitish points seen upon the skin in scarlatina. In a day or two, a gland on each side of the neck swelled and became painful. In this condition he continued, always wakeful and peevish, with slight fever, but nursing freely until the day before his death. On the morning of that day he had less fever, and his look was brighter; at night his fever increased.—The eruption had disappeared. He slept throughout the night unusually well, snoring loudly, until about 3 A. M., when he became restless. At 5 A. M. he began to breath badly, and at 9 A. M. his breathing was high and laborious, with mucous rattle, and he had no pulse; of this near approach of sinking, there had appeared no evidence the evening before. The chest sounded well on percussion. He lingered in this state until half past 4 P. M. and died without convulsions.

An elder sister and brother were sick at the same time. The former had very similar symptoms, and a similar eruption of the skin, but no cough. The boy had a regular attack of scarlatina, with slight sore throat.

*Autopsy.*—The body rather fat. The lungs were quite healthy, of a pale pink colour, crepitant throughout, and no serosity flowed from the divided extremities of the bronchi on squeezing them; on the lower lobe on the left lung was a purplish discoloration; and on incising it, a superficial degree of congestion existed for about a line in depth. The trachea was pale, but there was a slight degree of redness between the rings of the larger bronchi. I neglected to observe whether this was continued along the smaller ramifications. The thymus gland was enlarged. Its two cornua ascended up on the trachea to the distance of an inch above its bifurcation, lying in contact and completely covering it. The whole length of the cornua to where the gland begins to expand is an inch and a half, and the innominata artery is overlapped by them. The gland completely overlaps the heart, reaching to within one fourth of an inch of its apex, being two and one tenth inches at its widest part, and its whole length, including the cornua, four inches and a half. It adheres as usual, loosely to the pericardium. The heart is small; the walls of the left ventricle rather thick. The thymus, as is usual, is divided by a salcus into two lobes, of which the left is much the largest, being one and a quarter inches in width, and two and a half in length; the right, three fourths of an inch broad and two inches long. Its weight after immersion in spirit, 175 grains. The mucous membrane of the intestines was tumid, not vascular, every where easily removed by the nail or scalpel, and in some places partially removed by absorption. Several of Brunner's glands were in a state of considerable hypertrophy.

The symptoms, in this case, presented nothing attributable to the state of the thymus gland, nor do I learn that during life, he was subject to any attack of a spasmodic character. It is the sixth which I have witnessed; of these, four have already been published; the fifth will appear through another channel. Dr. Swett informs me that he has recently assisted at another post-mortem examination, in which enlargement of the thymus gland co-existed with slight broncho-pneumonia, which, with the case seen by Dr. Clements, makes eight cases of the disease met with in this city within a few years; a sufficient proof of the necessity for a more attentive examination of this interesting subject. It seems evident that children labouring under this congenital malformation, are, if they do not die suddenly, exceedingly liable to perish on the supervention of any slight degree of vascular excitement in their systems, by whatever cause induced. I would particularly remark that the patient did not die with the symptoms of cerebral disease; and that there did not exist disease enough within the chest to have caused death, unless the thymus gland be allowed to have exercised some agency.—*New York Hospital Reports, in N. Y. Journ. of Med. and Surg.*

---

*Case of Thymic Asthma.* By Dr. Bliss.—On the 12th of March, a female infant, aged six months, while playing with an attendant, who held it in her arms, suddenly threw the head back. The eyes became fixed, countenance livid, the extremites extended, rigid, and affected with a slight tremulous motion; which symptoms were followed with almost instant extinction of life.

About a month previous, I had been requested to visit the child, who had suffered two paroxysms similar to that which was the immediate cause of dath: one of which occurred the evening previous, and the other on the morning of the day I was requested to visit her. At this time she was affected with a deranged state of the bowels, frequent green watery stools, and she also had slight bronchial inflammation. These symptoms were relieved by remedies in the course of three or four days, and with the exception of an occasional slight cough and the recurrence in one instance of the symptoms above described, in which the countenance was remarkably livid, the mother represents the child to have been in apparent good health up to the moment of its death.

The circumstances of the case were such, as to induce me to solicit permission to examine the body; and Dr. Buck, at my request, made a dissection, the result of which, as furnished by his notes, is as follows:

*Dissection.*—The thymus gland measured four inches in length, and three in breadth, and consisted of a broad expanded portion below, that spread out over the heart, leaving only its apex uncovered. It adhered closely to the pericardium, and extended on either side to the roots of the lungs; its greatest thickness was three-fourths of an inch in the middle, at the edges it was thin and sharp. This portion sent a slender prolongation upwards as high as the inferior edge of the cricoid cartilage, that adhered by loose cellular tissues to the trachea and great vessels of the heart, and terminated in two, pointed, diverging crura that embraced the sides of the trachea. The substance of the gland was of the colour of the pancreas, and at its thickest part was of the same consistence. The mucous membrane lining the larynx and trachea was pale and healthy. The lungs were quite firm, and crepitated but little; their substance was red, tough, and exuded bu t little fluid. The heart was not examined. A small extent of the transverse colon was opened and found thickly scattered with swollen isolated glands.—*Ibid.*

